# Report of Columbia Hospital for Women

**Published:** 1874-02

**Authors:** 


Report of Columbia Hospital for Women, and Lying-in-Hospital, Washington, D. C. By J. Harry Thompson, A.M., M.D., Surgeon-in-Chief. Washington: Government Printing Office, 1873.—quarto, pp. 430.

This handsome volume from the Government press embodies not only a report of the management of the Columbia Hospital for Women, but presents, also, a creditable exposition of the present status of gynæcological medicine and surgery at the time of its issue. In an appendix is given a report of the dispensary attached to the hospital, including report of the Department of the Diseases of Women, by P. A. Ashford, M.D.; Department of Diseases of Children, by Samuel C. Busey, M.D.; and Department of Diseases of the Eye and Ear, by D. Webster Prentiss, M.D.

The volume is well illustrated by plates and wood cuts. Reports of cases, with methods of treatment are given of Ruptured Perineum. Dr. Thompson follows the method of the late Mr. I. Baker Brown, of London, with the following exceptions, viz: Dr. T. does not divide the sphincter ani, as practiced by Baker Brown, but in lieu thereof, "paralyzes the sphincter, as suggested by Prof. Van Buren, of New York." This is nothing more nor less,—as described by Dr. T.,—than the "rupturing of the sphincter," as practiced (and we believe claimed) by Dr. H. R. Storer, of Boston.

Dr. Thompson uses hard-rubber tubes in place of bits of elastic bougie, to support the lips of the denuded surface in firm apposition. In place of waxed twine he uses silver wire, and in the after treatment he does not constipate the bowels, after the method of Baker Brown, but secures a soluble daily evacuation.

In treating of Vesico-Vaginal Fistula, paying a just tribute of praise to the great American specialist, Dr. Marion Sims, the author makes the unguarded observation "for prior to his operations there was no record of a single successful case." (sic?) For the management of this malady he adopts the method of Sims. Operations for Vaginal Rectocele and Vaginal Cystocele follow, which present no points of special remark. The chapter on Diseases and Displacements of the Uterus is full and well considered. For Sub-involution of the Uterus Dr. T. uses constitutional measures, and local applications of carbolic acid (pure), chromic acid, and twenty gr. solution of nitrate silver to uterine cavity. In several cases the cervix was amputated.

"Is Carcinoma Uteri preventable? The conclusion to which I have arrived as the result of a moderately large experience is, that in the large majority of cases, the surgeon can, by judicious treatment, remove the exciting cause of the disease, and thus prevent an attack when the uterus is the organ threatened." Dr. Thompson maintains, that cancer is not constitutional in its origin, but the result of a slowly transpiring interstitial inflammation, dependent upon local irritation; that there is no specific cancer cell; that the probability of recurrence after ablation depends upon the richness of the part in lymphatics and the stage of the disease. He assigns as causes: whatever tends to excite slow interstitial inflammation of cervix at the time of life when the tissues are more prone to retrograde than progressive metamorphosis; acute inflammation allowed to degenerate into sub-acute or chronic form; repeated parturition and sub-involution; abortion. "I have seen very few cases where the history did not unmistakably point to this condition (chronic inflammation) as the exciting cause of the disease." *Ergo:* treat the chronic inflammation and, if need be, amputate the cervix early. His method is by the strong, blunt-pointed scissors; cut from the periphery towards the center, first on the right and then on the left side; "what little bleeding occurs is easily arrested by penciling the raw surface with a crystal of persulphate of iron." "It must not be understood that I recommend the amputation of the cervix uteri for chronic inflammation, as an operation to be resorted to in the early stages of that disease, but only when all other means of resolving the inflammation and induration have failed."

There are other portions of this interesting volume which we would be glad to notice, but time and space forbid.

It is to be obtained, by the favored few, through Members of Congress and the Department of the Interior.

